# Exosomes containing HIV protein Nef reorganize lipid rafts potentiating inflammatory response in bystander cells

**DOI:** 10.1371/journal.ppat.1007907

**Published:** 2019-07-25

**Authors:** Nigora Mukhamedova, Anh Hoang, Dragana Dragoljevic, Larisa Dubrovsky, Tatiana Pushkarsky, Hann Low, Michael Ditiatkovski, Ying Fu, Ryunosuke Ohkawa, Peter J. Meikle, Anelia Horvath, Beda Brichacek, Yury I. Miller, Andrew Murphy, Michael Bukrinsky, Dmitri Sviridov

**Affiliations:** 1 Baker Heart and Diabetes Institute, Melbourne, Australia; 2 Department of Immunology, Monash University, Melbourne, Australia; 3 Department of Microbiology, Immunology and Tropical Medicine, George Washington University, Washington, DC, United States of America; 4 Graduate School of Medical and Dental Sciences, Tokyo Medical and Dental University, Tokyo, Japan; 5 Department of Medicine, University of California San Diego, La Jolla, CA, United States of America; Miller School of Medicine, UNITED STATES

## Abstract

HIV infection has a profound effect on “bystander” cells causing metabolic co-morbidities. This may be mediated by exosomes secreted by HIV-infected cells and containing viral factors. Here we show that exosomes containing HIV-1 protein Nef (exNef) are rapidly taken up by macrophages releasing Nef into the cell interior. This caused down-regulation of ABCA1, reduction of cholesterol efflux and sharp elevation of the abundance of lipid rafts through reduced activation of small GTPase Cdc42 and decreased actin polymerization. Changes in rafts led to re-localization of TLR4 and TREM-1 to rafts, phosphorylation of ERK1/2, activation of NLRP3 inflammasome, and increased secretion of pro-inflammatory cytokines. The effects of exNef on lipid rafts and on inflammation were reversed by overexpression of a constitutively active mutant of Cdc42. Similar effects were observed in macrophages treated with exosomes produced by HIV-infected cells or isolated from plasma of HIV-infected subjects, but not with exosomes from cells and subjects infected with ΔNef-HIV or uninfected subjects. Mice injected with exNef exhibited monocytosis, reduced ABCA1 in macrophages, increased raft abundance in monocytes and augmented inflammation. Thus, Nef-containing exosomes potentiated pro-inflammatory response by inducing changes in cholesterol metabolism and reorganizing lipid rafts. These mechanisms may contribute to HIV-associated metabolic co-morbidities.

## Author summary

HIV infects only a limited repertoire of cells expressing HIV receptors. Nevertheless, co-morbidities of HIV infection, such as atherosclerosis, dementia, renal impairment, myocardial pathology, abnormal haematopoiesis and others, involve dysfunction of cells that can not be infected by HIV. These co-morbidities persist even after successful application of antiretroviral therapy, when no virus is found in the blood. Many co-morbidities of HIV have a common element in their pathogenesis, impairment of cholesterol metabolism. In this study we show that HIV protein Nef released from infected cells in extracellular vesicles is taken up by un-infected (‘bystander’) cells impairing cholesterol metabolism in these cells. This impairment causes formation of excessive lipid rafts, re-localization of the inflammatory receptors into rafts, and triggers inflammation. These mechanisms may contribute to HIV-associated metabolic co-morbidities. Our work demonstrates how a single viral factor released from infected cells into circulation may cause a pleiotropy of pathogenic responses.

## Introduction

HIV productively infects CD4^+^ T-cells, macrophages and related cells expressing CD4 receptor and CCR5 or CXCR4 co-receptors, but not other cell types that lack these molecules, and cannot replicate in tissues where susceptible cells are underrepresented. Nevertheless, clinical manifestations of HIV infection often involve dysfunction of cells and tissues which are not, and could not be, infected by HIV. HIV disease is associated with numerous co-morbidities, such as atherosclerosis, metabolic syndrome, myocardial pathology, abnormal adipose tissue, dementia, respiratory complications, abnormal haematopoiesis, and many others [1]. Paradoxically, many co-morbidities persist, albeit with reduced severity, even after successful application of antiretroviral therapy (ART), when no virus is detected in the blood and immunodeficiency is mitigated. One example is atherosclerosis and dyslipidaemia associated with HIV infection [2]. Pathogenesis of these co-morbidities involves vascular endothelial and smooth muscle cells as well as hepatic cells, none of which susceptible to HIV infection. Macrophages, which are also involved in pathogenesis of atherosclerosis, can be infected by HIV, however, the proportion of infected monocytes in blood and macrophages in tissues of ART-treated patients is too low to be a major driver of systemic atherosclerosis. One explanation of the systemic pathology in treated HIV infection is “bystander” effects. The effect of HIV infection on bystander cells has been described (for review see [3]) and was attributed to individual HIV proteins released from infected cells [4, 5] and taken up by uninfected cells. Nef (Negative Regulatory Factor), for example, is known to affect tissues through cytotoxicity, and other HIV proteins released from infected host cells may contribute to the systemic effects of the infection in various ways [6]. These effects can take place even in the context of effective anti-retroviral treatment due to ongoing expression of HIV proteins in long-living infected cells and HIV replication in viral reservoirs [5, 7]. Nef was found in blood of HIV-infected patients receiving ART [8, 9].

Many of the diverse co-morbidities of HIV disease have a common element that plays a prominent role in their pathogenesis, impairment of cholesterol metabolism. Cholesterol also plays a key role in the lifecycle of HIV, and HIV interacts with host cholesterol metabolism machinery [10]. We have previously identified the molecular mechanism by which HIV infection affects cholesterol metabolism [11]. HIV targets a pathway responsible for removal of excessive cholesterol from peripheral cells, reverse cholesterol transport pathway, and the key element of this pathway, lipid transporter ATP binding cassette transporter type A1 (ABCA1). We demonstrated the central role of the viral protein Nef in this activity: Nef inactivates host cell’s ABCA1 leading to reduction of cholesterol efflux and accumulation of intracellular cholesterol [11–13]. The concentration of free Nef in plasma of HIV-infected patients is, however, very low, and most Nef is secreted by HIV-infected cells in exosomes [9]. Exosomes were implicated in pathogenesis of HIV disease [14] and are increasingly considered an important way of cell-to-cell communication ensuring rapid and targeted delivery of the molecules from one cell to another [15]. The contribution of Nef-containing exosomes to systemic effects of HIV infection [16] and to pathogenesis of HIV-associated dementia [17] has been proposed. In this study, we investigated the effect of Nef-containing exosomes on uninfected macrophages and identified the mechanism that may play a key role in pathogenesis of several metabolic co-morbidities of HIV infection.

## Results

### Delivery of Nef by exosomes

We opted for the approach allowing investigation of the effects of Nef without interference of other HIV proteins because our previous studies demonstrated that Nef alone is fully responsible for the effects of HIV on cholesterol metabolism [11]. Nef-containing and control exosomes were generated in HEK293 cells transfected with, respectively, Nef_SF2_ or GFP. When indicated, cells were transfected with Nef tagged with GFP.

**Fig 1:**
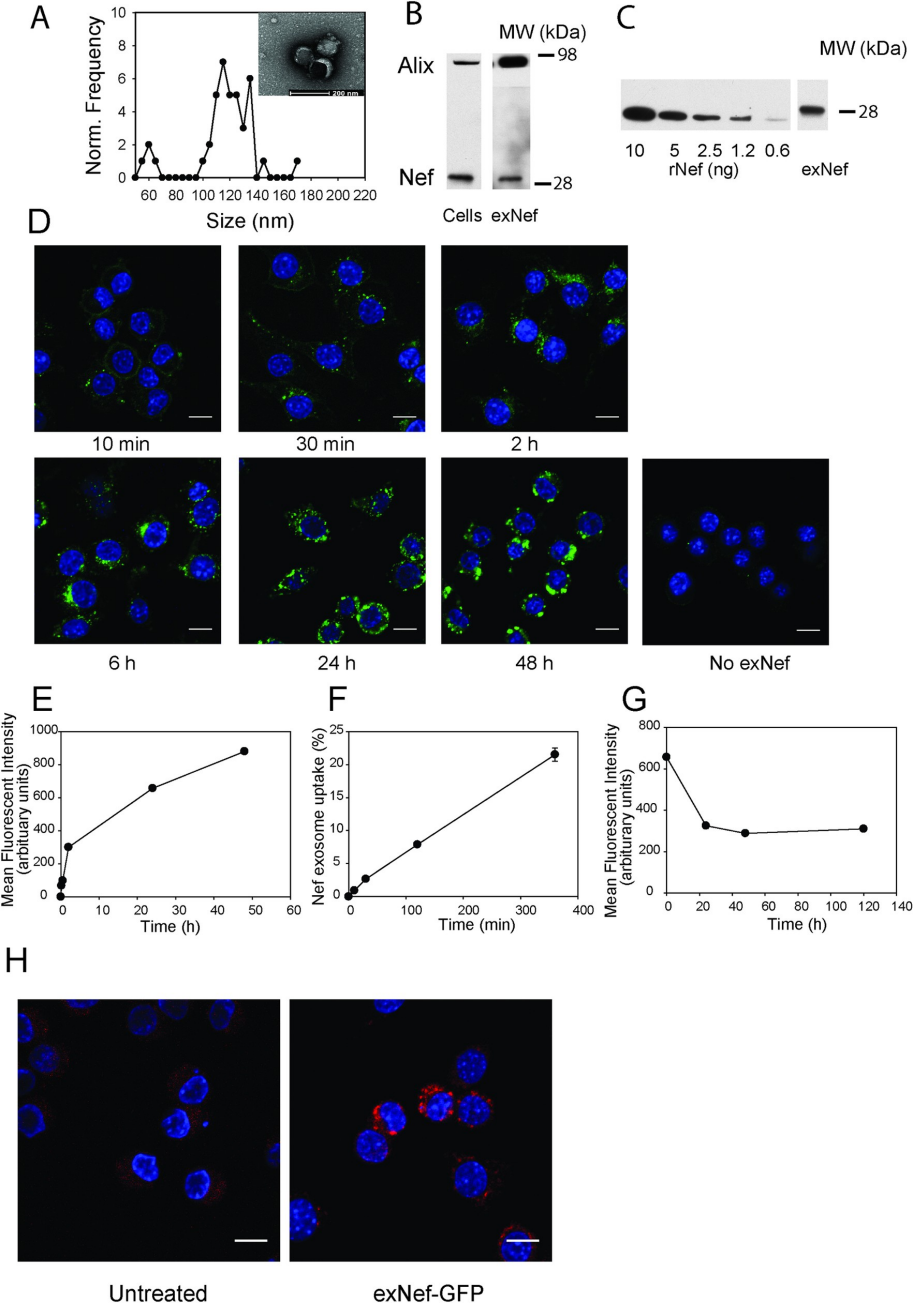
Nef-containing exosomes deliver Nef to macrophages. **A**—Size distribution of the extracellular vesicles secreted by HEK293 cells determined by EM; Inset–EM micrograph of the vesicles; bar– 200 nm. **B**—Western blot for the exosomal marker Alix and Nef in cells and exosomes (exNef); **C**—Western blot for the indicated amounts of rNef and in a typical preparation of exNef (10 μg of exosomal protein); **D, E**–Time-course of exosome uptake quantitated by confocal microscopy; **F**–Time-course of exosome uptake quantitated by fluorimetry; percentage of added exosomes that was taken up is shown; **G**–Cells were incubated with exosomes for 48 h, excess exosomes was washed out and cells incubated for the indicated periods of time in exosome-free medum; retained fluorscence of the exosome stain PKH67 was assessed using confocal microscopy; **H**–Visualisation of Nef-GFP inside the cells after exposure to exNef-GFP (5 μg/ml of exosomal protein) after staining with anti-GFP antibody. Scale bars– 10 μm.

Cell culture media was collected every 48 h for 8 days after transfection and extracellular vesicles (EV) were isolated by polyethylene glycol precipitation—based method. Size of the isolated EVs was analysed by electron microscopy; size distribution and a representative EM micrograph are shown in Fig 1A. Majority of the EVs were 120±20 nm, characteristic for exosomes [15]. The exosomal marker, Alix, was considerably enriched in EV preparations compared with its abundance in the cells (Fig 1B). We therefore termed the EVs used in this study exosomes, although it is recognized that exosomes in our preparations are likely to contain other types of EVs.

Exosomes isolated from Nef-expressing cells contained Nef (Fig 1B). To measure the concentration of Nef in Nef-containing exosomes (exNef), we generated a calibration curve using purified non-myristoylated recombinant Nef (rNef) produced using bacterial expression system, and quantitated the density of bands corresponding to recombinant and exosomal Nef using Western blot (Fig 1C). The Nef content of the exosomes showed considerable variability from one preparation to another, however, on average there was 0.5 ng Nef per 1 μg of total exosomal protein. In the experiments reported here, Nef concentration was determined for each individual preparation of exosomes. Total protein content of exosomes was measured, and GFP-containing exosomes (exGFP, control) were added at the same total protein concentration as Nef-containing exosomes.

To study the dynamics of exosome uptake, we labelled exosomes with fluorescent dye, PKH67, and incubated them with RAW264.7 macrophages for the indicated periods of time. Exosomes appeared in the cells after 10 min of incubation and were located in proximity to the plasma membrane (Fig 1D). Continuous incubation led to an almost linear increase in the abundance of labelled exosomes inside the cells for 6 h, followed by a slower uptake for at least another 42 h, with exosomes found across the cytosol (Fig 1D and 1E); there was no non-specific uptake of the dye (Fig 1D). Quantitation of exNef uptake by measuring total fluorescence intensity in the cells indicated that over 20% of added exosomes were taken up by cells after 6 h incubation (Fig 1F). When cells were loaded with exosomes for 48 h and unbound exosomes washed out, the abundance of labelled exosomes in the cells gradually decreased during 24 h, but up to 50% of exosomes originally taken up were retained by the cells for up to 120 h (Fig 1G). Staining of cells treated with exosomes containing GFP-tagged Nef (exNef-GFP) with anti-GFP antibody demonstrated that Nef-GFP could be detected in cells’ interior after 24 h incubation with the exosomes (Fig 1H). Thus, Nef-containing exosomes were rapidly and quantitatively taken up by macrophages, delivering Nef to the intracellular compartments.

It was previously suggested that exosomes produced by Nef-expressing cells contain Nef mRNA, which can be taken up and used to synthesize endogenous Nef in target cells [17]. Using RNAseq analysis we were unable to detect Nef mRNA in Nef-containing exosomes (deposited to GEO, accession #GSE122657).

### Nef-containing exosomes alter cholesterol metabolism

**Fig 2:**
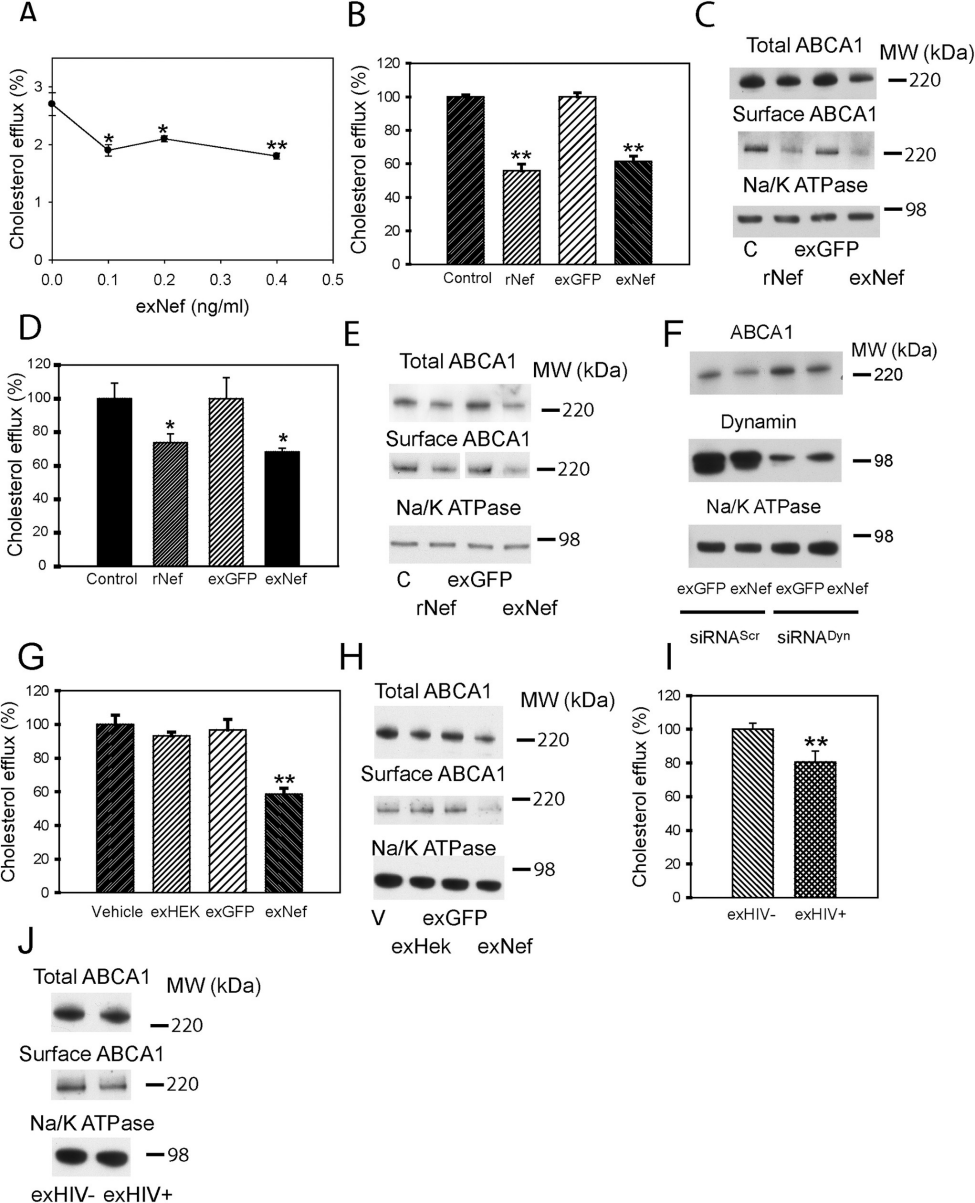
Exosomal Nef is more active than recombinant Nef. **A—**Dose-dependence of the effect of exNef on cholesterol efflux after 48 h exposure. *p<0.05, **p<0.01 *versus* control; **B**—Comparison of the effects of rNef (100 ng/ml) and exNef (0.4 ng/ml) on cholesterol efflux after 48 h exposure; **p<0.01 *versus* control; **C**—Comparison of the effects of rNef (100 ng/ml) and exNef (0.4 ng/ml) on total and cell-surface ABCA1 after 48 h exposure (Western blot); **D**—Comparison of the effects of rNef (100 ng/ml) and exNef (0.4 ng/ml) on cholesterol efflux from murine bone marrow derived macrophages after 48 h exposure; *p<0.05 *versus* control; **E**—Comparison of the effects of rNef (100 ng/ml) and exNef (0.4 ng/ml) on total and cell-surface ABCA1 in murine bone marrow derived macrophages after 48 h exposure (Western blot); **F**–The effect of silencing of Dynamin– 2 on the effect of exNef on the abundance of total ABCA1 (Western blot). Mean ± SEM are shown on graphs (n = 4); **G**—Comparison of the effects of vehicle, exosomes produced by un-transfected cells (exHEK), exGFP, and exNef (0.4 ng/ml) on cholesterol efflux after 48 h exposure; **p<0.01 *versus* all other bars; **H**—Comparison of the effects of vehicle, exosomes produced by un-transfected cells (exHEK), exGFP, and exNef (0.4 ng/ml) on total and cell-surface ABCA1 after 48 h exposure (Western blot); **I**—Comparison of the effects of exosomes isolated from plasma of uninfected donors (exHIV-) and HIV-infected donors undergoing treatment with ART (exHIV+) (4 μg/ml of exosomal protein, 48 h) on cholesterol efflux; **p<0.01 *versus* exHIV-; **J**—Comparison of the effects of exosomes isolated from plasma of uninfected donors (exHIV-) and HIV-infected donors undergoing treatment with ART (exHIV+) (4 μg/ml of exosomal protein, 48 h) on total and cell-surface ABCA1 (Western blot).

Our previous studies documented reduction of cholesterol efflux and ABCA1 abundance in macrophages infected with Nef-expressing HIV [11]. A similar effect was observed when recombinant myristoylated Nef was added to macrophages [13]. Here, we compared the effects of two forms of exogenously added Nef, exosomal Nef and myristoylated recombinant Nef produced in baculovirus expression system. Dose-dependence of the effect of exNef on cholesterol efflux from RAW264.7 macrophages is shown in Fig 2A, demonstrating a reduction of cholesterol efflux after a 48 h incubation with exNef at concentration as low as 0.1 ng/ml (3.3 nmol/L). ExGFP had no effect on cholesterol efflux at exosome concentrations equivalent to the highest concentration of exNef (Fig 2B and 2G). When we compared side-by-side the effects of rNef and exNef on cholesterol efflux from RAW264.7 cells after 48 h incubation, exNef caused the same level of reduction of cholesterol efflux as rNef at concentration 250-fold lower (exNef—0.4 ng/ml (13.2 nMol/L) *versus* rNef 100 ng/ml (3.3 μMol/L, minimal effective concentration)) (Fig 2B). Correspondingly, exNef caused a similar reduction in the abundance of total and cell-surface ABCA1 at a 250-fold lower concentration compared to rNef (Fig 2C). To ensure that the findings with exNef were not specific to RAW264.7 cells, we repeated these experiments using primary cells, murine bone marrow derived macrophages (BMDM). Similar to RAW264.7 cells, exNef was effective in reducing cholesterol efflux (Fig 2D) and abundance of total and cell-surface ABCA1 (Fig 2E) in BMDM at concentrations 250-fold lower than concentrations of rNef that caused similar effects. The higher functional efficiency of exNef is most likely explained, at least partially, by a much higher efficiency of the uptake of exosomes by target cells via endocytic pathways, in comparison to rNef that does not have a specific receptor [18]. Indeed, inhibition of endocytosis by silencing Dynamin-2, a common element of several endocytic pathways, partially inhibited the effects of exNef on the abundance of ABCA1 (Fig 2F), indicating involvement of active endocytic pathways in the exNef activity. To ensure that the exosomes themselves do not produce similar functional effects, we compared the effects of exNef and exGFP with that of exosomes produced by un-transfected cells and of the vehicle (Fig 2G and 2H). Only Nef-containing exosomes reduced cholesterol efflux and abundance of total and cell-surface ABCA1, supporting the notion that the effects on cholesterol efflux and ABCA1 were caused by Nef. Finally, we compared exosomes isolated from plasma of uninfected volunteers and from HIV-infected subjects. Plasma was pooled from 4 uninfected and from 4 HIV-infected subjects undergoing treatment with ART and described in our recent study [19]. Exosomes from HIV-infected, ART treated donors (HIV+) reduced cholesterol efflux as well as abundance of total and cell-surface ABCA1 compared to exosomes from uninfected individuals (Fig 2I and 2J).

**Fig 3:**
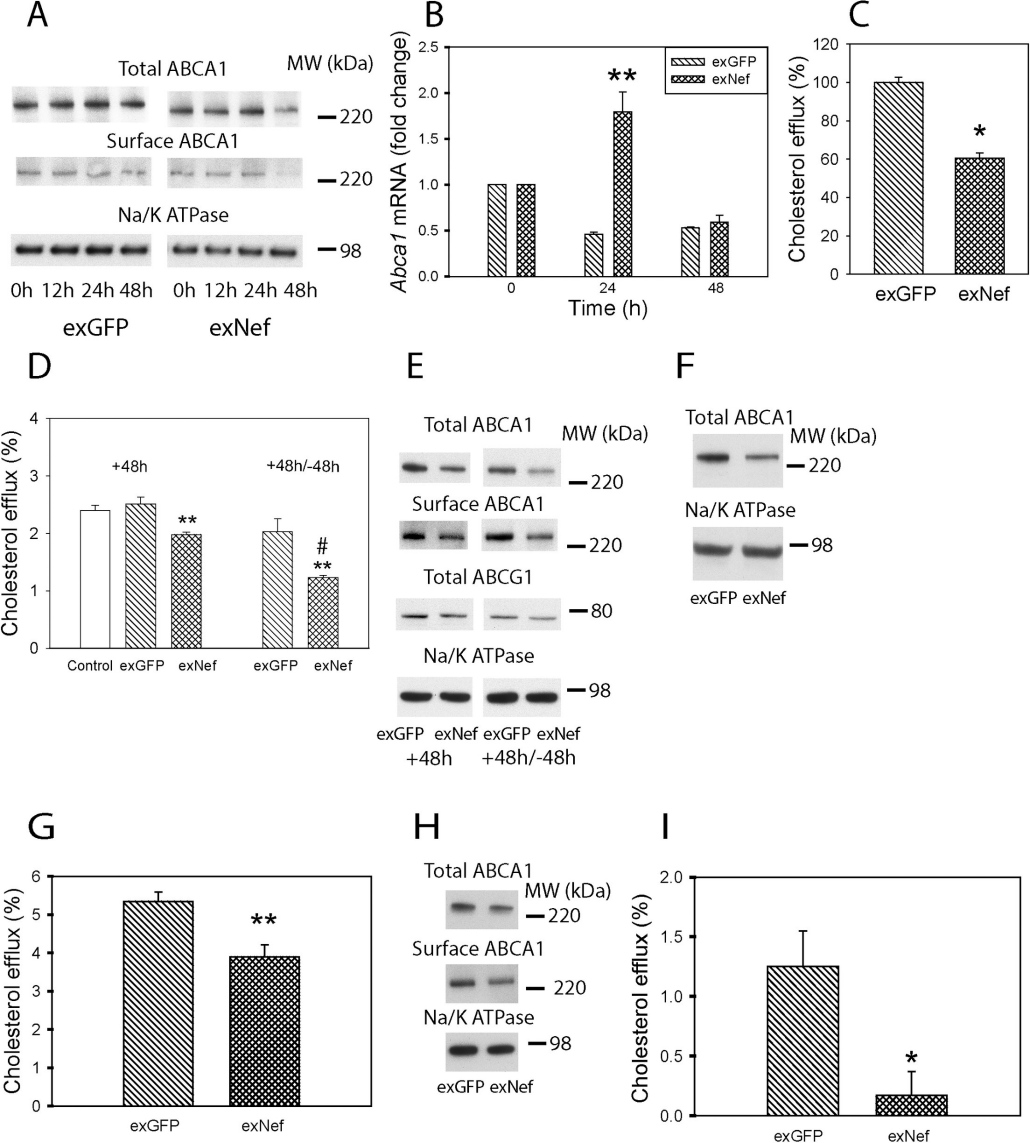
ExNef modify cellular cholesterol metabolism. A—Time-course of the effect of exNef (0.4 ng/ml) on the abundance of total and cell-surface ABCA1 (Western blot); B—Abundance of *Abca1* mRNA (qRT-PCR) after exposure to exGFP or exNef (0.4 ng/ml); **p<0.01 *versus* exGFP; C–Average change in cholesterol efflux after exposure of cells to exGFP or exNef (0.4 ng/ml) for 48 h (*p<0.05, n = 6); D—Cholesterol efflux after exposure of cells to exGFP or exNef (0.4 ng/ml) for 48 h (left) or for 48 h followed by incubation without exosomes for another 48 h (right); #p<0.05 *versus* exNef 48 h, **p<0.01 *versus* exGFP; E—Abundance of total ABCG1 and total and cell-surface ABCA1 after exposure of cells to exGFP or exNef (0.4 ng/ml) for 48 h or for 48 h followed by incubation without exosomes for another 48 h (Western blot); F—Abundance of ABCA1 after co-cultivation of RAW264.7 cells with Nef or GFP producing HEK293 cells for 48 h (Western blot); G—Comparison of the effects of exGFP and exNef (0.4 ng/ml) on cholesterol efflux from THP-1 human monocyte-macrophages after 48 h exposure; **p<0.01 *versus* exGFP; H—Comparison of the effects of exGFP and exNef (0.4 ng/ml) on the abundance of total and cell surface ABCA1 in THP-1 human monocyte-macrophages after 48 h exposure (Western blot); I—Comparison of the effects of exGFP and exNef (0.4 ng/ml) on cholesterol efflux from human monocyte derived macrophages after 48 h exposure. *p<0.05 *versus* exGFP. Mean ± SEM are shown on graphs.

Next, we investigated the dynamics of the effect of exNef on cellular cholesterol metabolism. ExNef reduced the abundance of both total and cell-surface ABCA1 in RAW264.7 cells in a time-dependent manner (Fig 3A). After a 48 h exposure to exNef, the abundance of total ABCA1 was reduced by 42±1%, and that of cell-surface ABCA1—by 40±1% (mean ± SEM, p<0.01; n = 3). Expression of *Abca1* was increased after a 24 h exposure to exNef, but there was no statistical difference in *Abca1* expression after 48 h of exposure between exGFP and exNef (Fig 3B). The discrepancy between ABCA1 abundance and gene expression indicates that regulation of ABCA1 abundance by exNef mainly occurs at a post-transcriptional level, as we suggested for endogenously expressed Nef [20]. On average, cholesterol efflux to apoA-I was reduced by 40±3% (mean ± SEM, p<0.05; n = 6) after 48 h exposure to exNef (Fig 3C).

To investigate the duration of the Nef effects, we incubated cells with exNef for 48 h, then removed exNef and incubated cells for further 48 h in the exosome-free media. Unexpectedly, the effects of exNef on cholesterol efflux (Fig 3D) and ABCA1 abundance (Fig 3E) became even stronger after removal of exNef from the media, indicating that Nef delivered by exosomes remains active within the cells for at least 48 h. The abundance of another cholesterol transporter, ATP Binding Cassette Transporter G1 (ABCG1), was not affected by exNef (Fig 3E). We also tested an option of constant delivery of exosomes by co-culturing GFP- or Nef-transfected HEK293 cells with RAW264.7 cells in chambers of a transwell for 48 h. Again, the abundance of total ABCA1 was reduced in RAW264.7 cells exposed to the media produced by Nef-expressing compared to GFP-expressing HEK293 cells (Fig 3F). Finally, we tested the effects of exNef on human macrophages. Exposure of differentiated THP-1 cells to exNef for 48 h effectively reduced cholesterol efflux (Fig 3G) and abundance of both total and cell surface ABCA1 (Fig 3H) in these cells relative to cells treated with exGFP. Cholesterol efflux from exNef-treated human monocyte-derived macrophages (MDM) was also significantly reduced (Fig 3I).

Expression of Nef may change the miRNA profile of exosomes, including abundance of miRNAs involved in regulation of cholesterol metabolism [21, 22]. However, sequencing miRNAs in exosomes yielded only 4 miRNAs that were reliably different in Nef-containing exosomes (S1 Dataset, deposited to GEO, accession #GSE122657): hsa-mir-6882, hsa-mir-4786, hsa-mir-1255a and hsa-mir-381. None of these miRNAs targets ABCA1 or has a known function in lipid metabolism.

### Nef-containing exosomes reorganize lipid rafts

**Fig 4:**
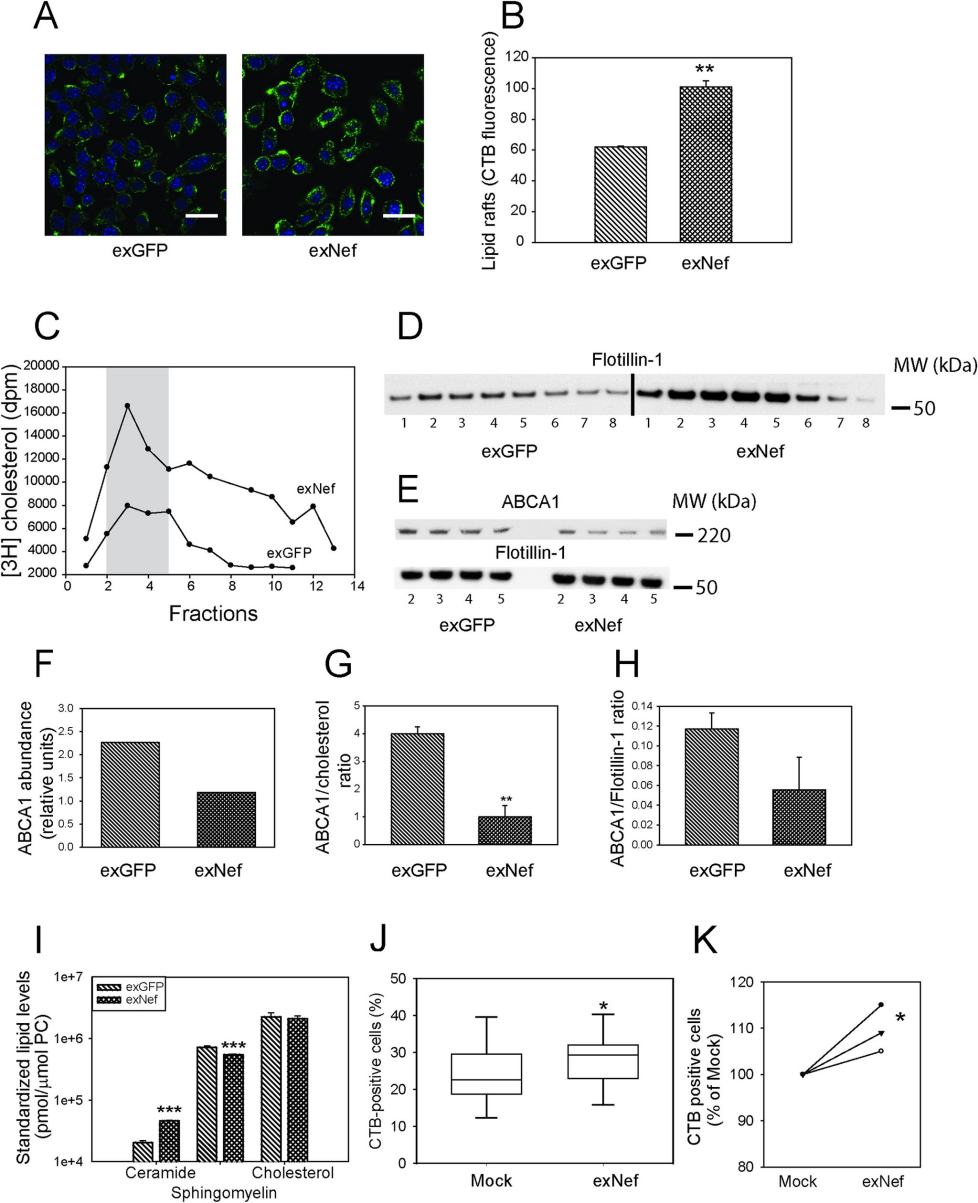
ExNef reorganize lipid rafts. **A**–The effect of exNef (0.4 ng/ml, 48 h) on the abundance of lipid rafts in RAW 264.7 cells (cholera toxin subunit B (CTB) staining, confocal microscopy; Scale bars– 10 μm. **B**–Quantitation of the effect of exNef on the abundance of lipid rafts in RAW 264.7 cells by confocal microscopy (CTB staining); **p<0.01 *versus* exGFP; **C**—Amount of [^3^H]cholesterol in fractions of density gradient centrifugation (from top to bottom) of plasma membranes from exGFP or exNef treated RAW 264.7 cells. **D–**Abundance flotillin-1 in fractions of density gradient centrifugation (from top to bottom) of plasma membranes from exGFP or exNef treated RAW 264.7 cells (Western blot). **E**—Abundance of ABCA1 in raft fractions from exGFP or exNef treated RAW 264.7 cells (Western blot). **F–**Quantitation (densitimetry) of cummulative abundance of ABCA1 in raft fractions. **G–**Ratio of ABCA1 abundance to [^3^H]cholesterol counts in raft fractions; **p<0.01 *versus* exGFP; **H–**Ratio of abundancies of ABCA1 to flotillin-1 in raft fractions. **I–**Concentration of cholesterol, total ceramides and total sphyigomielin in isolated lipid rafts; ***p<0.001 *versus* exGFP **J—**Quantitation of the effect of exNef on the abundance of lipid rafts in human monocyte derived macrophages by flow cytometry (CTB staining); box plot of n = 6 is shown, *p<0.05. **K—**Quantitation of the effect of exNef on the abundance of lipid rafts in CD4+ T-cells by flow cytometry (CTB staining); n = 3, *p<0.05.

One consequence of inactivation of ABCA1 and suppression of cholesterol efflux is an increase in the abundance of lipid rafts [23, 24]. Lipid rafts play a prominent role in pathogenesis of many diseases, from cardiovascular disease and inflammation to dementia [25, 26]. We previously demonstrated that intracellular expression of Nef results in increased abundance of rafts in macrophages [12]. Incubation of murine macrophages with exNef for 48 h resulted in a significant increase in the abundance of rafts determined by binding of cholera toxin subunit B (CTB) and assessed by confocal microscopy (Fig 4A and 4B). We then labelled cells with [^3^H]cholesterol under conditions allowing for equilibration of [^3^H]cholesterol in cellular cholesterol pools, and isolated rafts by density gradient centrifugation of the plasma membrane fraction. Distribution of [^3^H]cholesterol along the density gradient fractions is shown in Fig 4C, and distribution of flotillin-1 (marker of rafts) is shown in Fig 4D. We designated fractions 2–5 as rafts because they contained the highest concentrations of [^3^H]cholesterol and flotillin-1 (Fig 4C and 4D). Raft fractions from cells treated with exNef contained more cholesterol and more flotillin-1 than corresponding fractions from cells treated with exGFP (Fig 4C and 4D). To estimate the concentration of ABCA1 per raft we analysed levels of ABCA1 in gels where fractions were loaded at the same flotillin-1 concentration (Fig 4E). ABCA1 levels (Fig 4F) as well as the ratios of ABCA1 to [^3^H]cholesterol (Fig 4G) and ABCA1 to flotillin-1 (Fig 4H) were reduced in rafts of exNef-treated cells, indicating that these cells contain less ABCA1 per raft. We next performed lipidomic analysis of isolated lipid rafts. Concentrations of lipids critical for the raft structure are shown in Fig 4I; the results of analysis of over 700 lipid species in rafts are presented in the S2 Dataset. When analysed relative to phosphatidylcholine level (*i*.*e*. on a per raft basis), rafts isolated from cells treated with exNef contained higher concentration of total ceramides and slightly lower concentration of total sphingomyelin when compared to rafts isolated from cells treated with exGFP; there was no difference in raft cholesterol concentration. Finally, we analysed rafts in exNef-treated human MDM from eight donors. The abundance of rafts, measured by flow cytometry after CTB staining was significantly increased in MDM treated with exNef relative to cells treated with control exosomes (p<0.03, n = 6) (Fig 4J). The abundance of rafts was also increased in CD4+ T-cells (p<0.05, n = 3), although the effects were smaller, presumably because T-cells, compared to MDM, have lower abundance of ABCA1 [12], the main target of exNef. Collectively, these findings indicate that exNef increases the abundance of lipid rafts, changes lipid raft composition and reduces the abundance of ABCA1 in rafts.

### Nef-containing exosomes potentiate inflammation via re-localization of TLR4 and TREM-1 to lipid rafts

**Fig 5:**
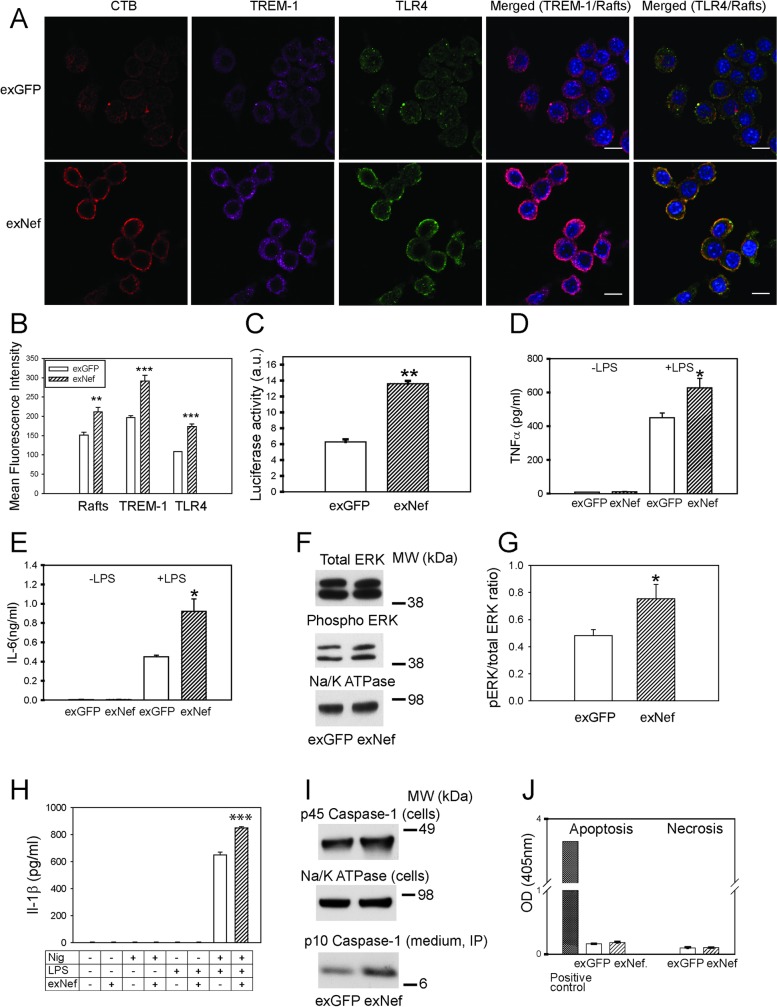
ExNef potentiate inflammatory signalling cascade *via* re-localization of TLR4 and TREM-1 to lipid rafts. **A**–The effect of exNef (0.4 ng/ml, 48h) on the abundance of lipid rafts and re-localization of TLR4 and TREM-1 to the plasma membrane in RAW264.7 macrophages. Left column, CTB staining; second column, anti-TREM-1 staining; third column anti-TLR4 staining; fourth column–merge TREM-1/rafts, right column–merge TLR4/rafts. Scale bars 10 μm; **B**–Quantitation of the effect of exNef (0.4 ng/ml, 48 h) on the abundance of lipid rafts, TREM-1 and TRL4 at the plasma membrane. Mean ± SEM are shown; **p<0.01, ***p<0.001 versus exGFP; **C**–Bio-assay for inflammatory cytokines secreted by macrophages treated with exNef (0.4 ng/ml, 48 h) after stimulation with LPS (100 ng/ml of LPS for 18 h). Luminescence produced by SVEG/VCAM endothelial cells (stably transfected with luciferase under VCAM-1 promoter) is shown (see Methods for details). **p<0.001. **D**–The effect of exNef on TNFα secretion by human monocyte derived macrophages with or without stimulation with 1 ng/ml of LPS for 24 h; *p<0.05. **E**–The effect of exNef on IL-6 secretion by human monocyte derived macrophages with or without stimulation with 1 ng/ml of LPS for 24 h; *p<0.05. **F**–The effect of exNef (0.4 ng/ml, 48 h) on the abundance of total and phosphorylated ERK1/2 in RAW 264.7 macrophages (Western blot); **G**–Ratio of phosphorylated to total ERK1/2 (Western blots, n = 3, *p<0.05); **H-** Release of Il-1β from BMDM pre-treated with exNef (0.4 ng/ml, 48 h) and treated with LPS (100 ng/ml, 4 h)) and Nigericin (5μM, 3 h). Control cells were treated with exGFP; n = 4, ***p<0.001. **I**–The effect of exNef (0.4 ng/ml, 48 h) on the abundance of pro-caspase-1 in the BMDM cell lysate and of the cleaved p10 form immunoprecipitated from the cell culture medium (Western blot); **J**–The effect of exNef (0.4 ng/ml, 48 h) on apoptosis and necrosis in RAW 264.7 macrophages (TUNEL assay).

Lipid rafts host many receptors involved in inflammatory responses and play a key role in regulation of inflammation [26]. To investigate if increased abundance of lipid rafts caused by exNef affects innate inflammatory responses of macrophages, we tested the effect of exNef on intracellular distribution of TLR4, a key inflammatory receptor, and TREM-1, an important amplifier of innate immune responses [27, 28] and contributor to the foam cell formation and atherogenesis [29]. Treatment of macrophages with exNef caused re-distribution of TREM-1 and TLR4 to the plasma membrane, dramatically increasing abundance of TLR4, TREM-1 and rafts on the cell surface, and augmented co-localization of TLR4 and TREM-1 with rafts (Fig 5A and 5B). There was a considerable correlation between the abundance of rafts and TREM-1 at the plasma membrane (r = 0.73±0.09, p<0.001) and high level of co-localization between CTB and TREM-1 (95±2.3%).

Next, we tested the effect of exNef on inflammatory responses by utilizing a bio-assay to estimate cytokine production [30]. RAW264.7 cells were treated for 48 h with exNef or exGFP and activated with LPS (24 h); medium was collected, exosomes removed by ultracentrifugation, and supernatant used to treat murine vascular endothelial cells stably expressing luciferase under the VCAM-1 promoter (SVEG/VCAM). Treatment of RAW 264.7 cells with exNef resulted in an increased release of factors activating VCAM-1 in endothelial cells (Fig 5C). Consistent with findings in murine cells, treatment of human MDM with exNef for 48 h caused a significant increase of secretion of TNFα (Fig 5D) and IL-6 (Fig 5E) after stimulation with LPS; unstimulated secretion of both TNFα and IL-6 in these cells was very low and unaffected by exNef (Fig 5D and 5E).

To confirm that redistribution and activation of TLR4 results in downstream inflammatory responses, we tested the effect of exNef on phosphorylation of ERK1/2. While there was no difference in total ERK1/2 abundance between exGFP and exNef treated cells, the abundance of phosphorylated (active) ERK1/2, and consequently the ratio of phosphorylated to total ERK1/2, were significantly higher in cells treated with exNef compared to cells treated with exGFP (Fig 5F and 5G). Next, we tested the effect of exNef on activation of NLRP3 inflammasome. In these experiments formation of inflammasome in BMDM isolated from C57Bl/6 mice was primed by treatment with LPS and then the release of interleukin IL-1β was induced by Nigericin. The amount of IL-1β released from cells pre-treated with exNef was statistically significantly higher than that from cells pre-treated with exGFP (Fig 5H). Furthermore, pre-treatment with exNef increased cleavage of pro-caspase-1 with release of proteolytically cleaved p10 form to the cell culture medium (Fig 5I). These experiments indicate that exNef potentiates a signalling cascade originating from TLR4 and resulting in NLRP3 inflammasome activation and release of IL-1β.

Finally, we assessed the possible toxicity of exNef. Neither exGFP nor exNef caused an appreciable necrosis or apoptosis in murine macrophages (Fig 5J).

### Nef-containing exosomes regulate rafts and inflammation via Cdc42 axis

**Fig 6:**
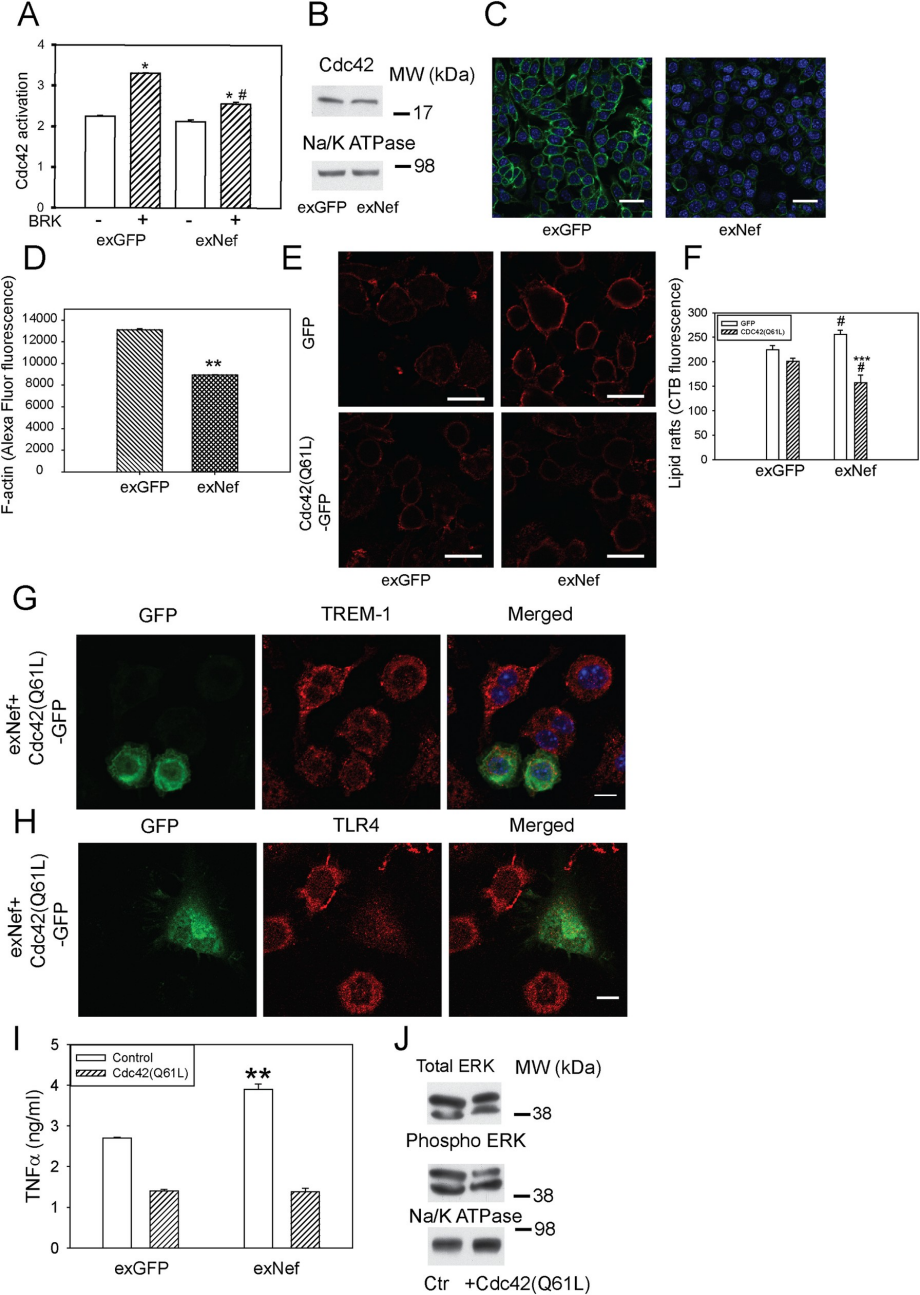
ExNef reorganize lipid rafts and potentiate inflammation *via* the ABCA1-Cdc42-actin axis. A—The effect of exNef (0.4 ng/ml) on activation of Cdc42 by bradykinin (BRK, 100 ng/ml). Cdc42 activity was assessed by G-LISA, the assay detects concentration of GTP-bound (active) Cdc42. Mean ± SEM is shown; *p<0.05 *versus* non-activated cells; ^#^p<0.01 *versus* activated cells treated with exGFP; (n = 4); B–The effect of exNef (0.4 ng/ml) on the abundance of total Cdc42 (Western blot). C–The effect of exNef (0.4 ng/ml, 48 h) on the abundance of F-actin (Alexa Fluor 488 Phalloidin staining); Scale bars– 10 μm. D–Quantitation of the effect of exNef on the abundance of F-actin. Mean ± SEM is shown; **p<0.01; E–The effect of transfection of cells with with GFP (top row) or Cdc42(Q61L)-GFP (bottom row) before exposure to exNef (0.4 ng/ml, 24 h) on the abundance of rafts (CTB staining, confocal microscopy); Scale bars– 10 μm. F–Quantitation of the effect of transfection of cells with GFP or Cdc42(Q61L)-GFP before exposure to exNef (0.4 ng/ml, 48 h) on the abundance of rafts (CTB staining, confocal microscopy); ***p<0.001 *versus* GFP-transfected cells; ^#^p<0.05 *versus* corresponding exGFP-treated cells. G–The effect of transfection of cells with with Cdc42(Q61L)-GFP before exposure to exNef (0.4 ng/ml, 24 h) on the localization of TREM-1; left panel, Cdc42-GFP (transfected cells), middle panel, TREM-1, right panel, merge. Scale bars 10 μm; H–The effect of transfection of cells with with Cdc42(Q61L)-GFP before exposure to exNef (0.4 ng/ml, 24 h) on the localization of TLR4; left panel, Cdc42-GFP (transfected cells), middle panel, TLR4, right panel, merge. Scale bars 10 μm. I—The effect of transfection of cells with with Cdc42(Q61L)-GFP before exposure to exNef (0.4 ng/ml, 48 h) and stimulation with LPS (100 ng/ml, 18 h) on secretion of TNFα. Means ±SEM is shown; **p<0.01 *versus* exGFP. J—The effect of transfection of cells with Cdc42(Q61L) on the abundance of phosphorylated ERK1/2 in RAW 264.7 macrophages treated with exNef (0.4 ng/ml, 48 h) (Western blot).

To gain further insight into the mechanism of the effect of Nef on the abundance of lipid rafts and inflammation, we tested a hypothesis originally proposed by Nofer et al. [31, 32] and validated in later studies [33], that a reciprocal connection between ABCA1 abundance and lipid rafts is maintained through activation of the small GTPase Cdc42, polymerization of actin, and subsequent negative regulation of raft abundance [34]. Thereby, by reducing the abundance of ABCA1, exNef may dampen the Cdc42 activation, reduce actin polymerization, and increase lipid raft abundance [30, 34]. Indeed, activation of Cdc42 by bradykinin was impaired in cells treated with exNef for 48 h (Fig 6A), whereas the overall abundance of Cdc42 was not affected (Fig 6B). The abundance of filamentous actin (F-actin) was reduced dramatically in cells treated with exNef (Fig 6C and 6D). To overcome an impairment in Cdc42 activation caused by exNef, we transfected cells with constitutively active mutant of Cdc42, Cdc42(Q61L)-GFP [35]. Overexpression of Cdc42(Q61L)-GFP reduced lipid raft abundance in macrophages; the abundance of rafts in cells treated with exNef was reduced below their level in cells treated with exGFP (Fig 6E and 6F).

Next, we tested the effects of exNef on distribution of TREM-1 and TLR4 in RAW 264.7 macrophages after transfecting cells with Cdc42(Q61L)-GFP. As expected, in mock-transfected control cells treated with exNef (cells negative for GFP) a considerable proportion of TREM-1 (Fig 6G) and TLR4 (Fig 6H) was localized to the plasma membrane. However, when Cdc42(Q61L)-GFP was overexpressed (cells positive for GFP), The abundance of TREM-1 and TLR4 on the plasma membrane was significantly reduced (Fig 6G and 6H). Furthermore, while secretion of TNFα after stimulation of cells with LPS was higher after treatment of cells with exNef, in cells transfected with Cdc42(Q61L)-GFP secretion of TNFα was reduced to the level of control (exGFP-treated) Cdc42(Q61L) expressing cells (Fig 6I). Reduction of TNFα secretion in exNef treated cells transfected with Cdc42 (Q61L)-GFP was accompanied with reduction in phosphorylation of ERK1/2, total ERK1/2 was not affected (Fig 6J). Thus, it appears that exNef, likely through its effects on ABCA1, impairs activation of Cdc42 leading to reduction of actin polymerization, increased abundance or lipid rafts and potentiated inflammatory response.

### Nef-containing exosomes modify lipid rafts and potentiate inflammation in vivo

**Fig 7:**
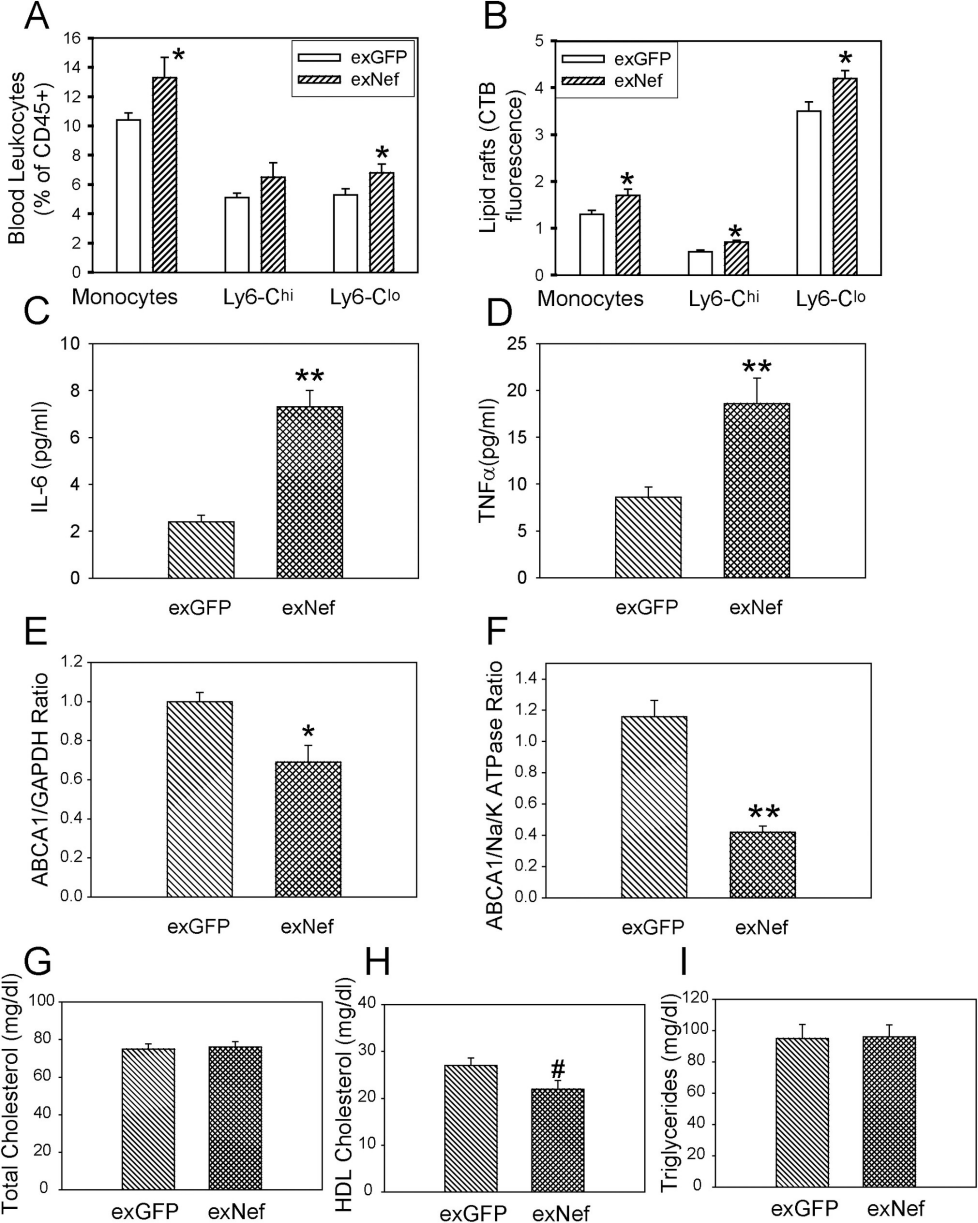
ExNef modify lipid rafts and potentiate inflammation *in vivo*. C57BL/6 mice were administered either exNef or exGFP (2μg, I.V.) 3 times a week, for a period of 2 weeks. **A–**Proportion of monocytes in blood (n = 9 per group), *p<0.05 *versus* exGFP; **B—**Flow cytometry analysis of raft abundance (CTB binding) in blood monocytes (n = 9 per group); *p<0.05 *versus* exGFP; **C**–Plasma IL-6 content (n = 8 per group); **p<0.01 *versus* exGFP; **D**–Plasma TNFα content (n = 8 per group); **p<0.01 *versus* exGFP; **E**—ABCA1 abundance in the liver homogenate determined by Western blot (n = 8 per group); *p<0.05 *versus* exGFP; **F**—ABCA1 abundance in the peritoneal macrophages determined by Western blot (n = 6 per group); **p<0.01 *versus* exGFP; **G**–Plasma total cholesterol content (n = 8 per group); **H**–Plasma HDL cholesterol content (n = 8 per group); #p = 0.05 *versus* exGFP, **I**–Plasma triglyceride content (n = 8 per group). Mean ± SEM are shown.

To confirm our findings in an *in vivo* setting, we injected exNef or exGFP intravenously into C57Bl/6 mice. Exosomes (2 μg protein per injection) were injected 3 times per week for 2 weeks; blood and tissues were then collected for analysis. The proportions of monocytes and in particular of a Ly6-C^lo^ monocyte subset (patrolling monocytes) were elevated in mice treated with exNef (Fig 7A). The abundance of rafts in total monocytes and in Ly6-C^hi^ (inflammatory monocytes) and Ly6-C^lo^ subsets was measured by flow cytometry. We found increased abundance of rafts in these cells after treatment of animals with exNef as compared to treatment with exGFP (Fig 7B). This was accompanied with elevation of the plasma levels of IL-6 (Fig 7C) and TNFα (Fig 7D). Next, we analysed the effect of exNef on ABCA1 abundance in the liver and peritoneal macrophages. The abundance of ABCA1 in liver and peritoneal macrophages was reduced by 30% and 65%, respectively, in mice treated with exNef when compared to treatment with exGFP (Fig 7E and 7F). There was no effect of exNef infusion on plasma total cholesterol or triglyceride content, however, there was a borderline reduction (20%, p = 0.05) in plasma HDL cholesterol content (Fig 7G–7I). We next performed lipidomics analysis of plasma samples (S3 Dataset). We found no difference in the abundance of lipid species between plasma of mice injected with exNef *versus* exGFP-injected mice. Thus, the main effect of exNef *in vivo*, at least in the short term, was on cellular, rather than systemic lipid metabolism.

### Nef-containing exosomes from HIV-infected cells and isolated from plasma of HIV-infected subjects modify lipid rafts and potentiate inflammation

**Fig 8:**
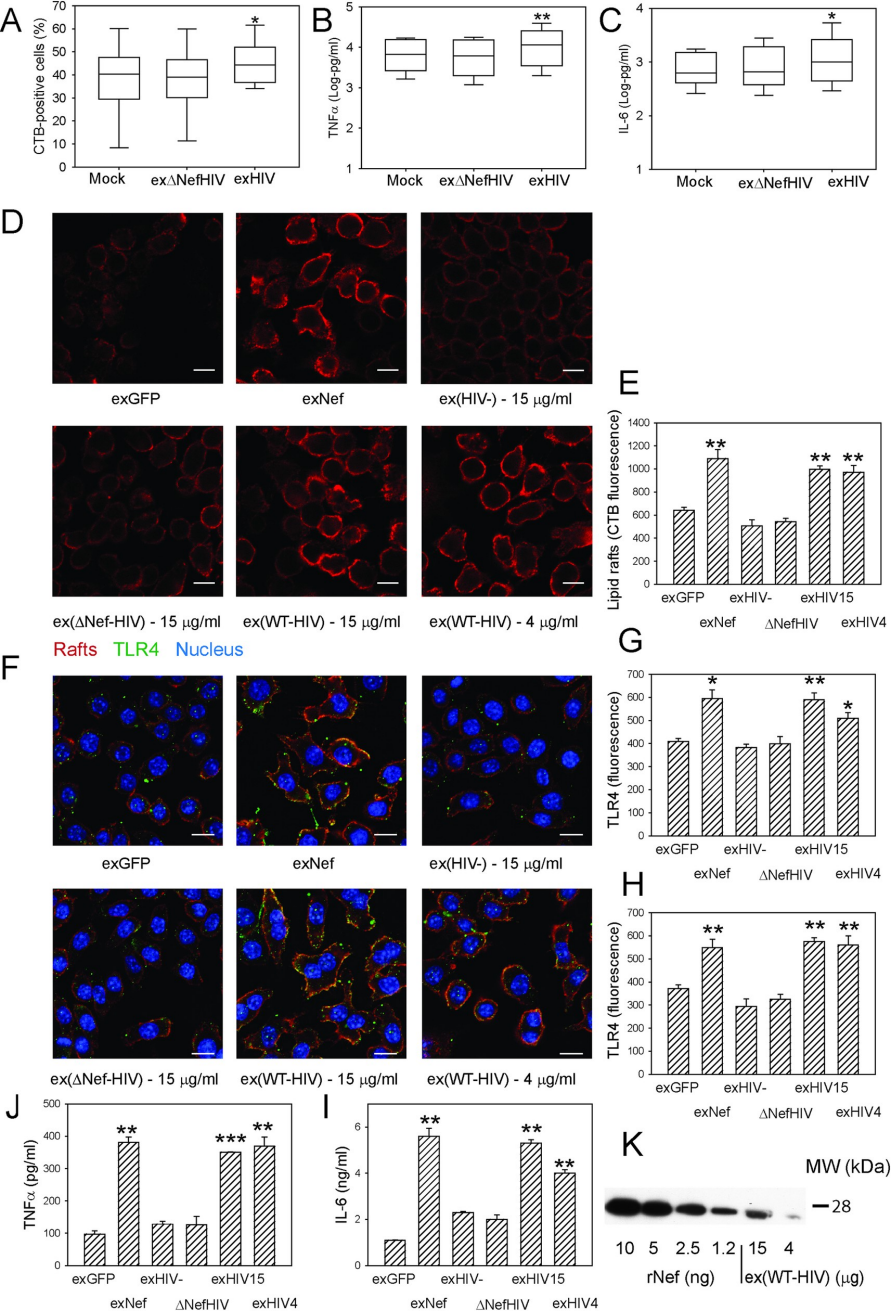
Exosomes from HIV-infected cells and plasma of HIV-infected subjects modify rafts and potentiate inflammation. **A–**The effect of exosomes produced by human monocyte derived macrophages (MDM) infected with HIV (exHIV) or ΔNefHIV (exΔNefHIV**)** on the abundance of rafts in MDM measured with flow cytometry; 48 h incubation; box plot of n = 6 (six separate experiments with MDM from 6 different donors) is shown; *p<0.05 *versus* both Mock and exΔNefHIV; **B–**The effect of exosomes produced by human monocyte derived macrophages infected with HIV (exHIV) or ΔNefHIV (exΔNefHIV**)** on secretion of TNFα by MDM over 48 h; box plot of n = 6 is shown; **p<0.01 *versus* both Mock and exΔNefHIV; **C–**The effect of exosomes produced by human monocyte derived macrophages infected with HIV (exHIV) or ΔNefHIV (exΔNefHIV**)** on the secretion of IL-6 over 48 h incubation. N = 6, *p<0.05 *versus* both Mock and exΔNefHIV; **D**–The effect of exNef, exGFP and exosomes isolated from plasma of subjects infected with HIV (ex(WT-HIV)) or infected with Nef-deficient strain of HIV (ex(ΔNef-HIV)) or uninfected (ex(HIV-)) (15 μg/ml of exosomal protein except when 4 μg/ml is indicated, 48h) on the abundance of lipid rafts in RAW264.7 macrophages. Scale bars 10 μm; **E**–Quantitation of the effect of exosomes (as in A)) on the abundance of lipid rafts. Mean ± SEM are shown; **p<0.01 *versus* exGFP and ex(HIV-). **F**–The effect of exNef, exGFP and exosomes isolated from plasma of subjects infected with HIV (ex(WT-HIV)) or infected with Nef-deficient strain of HIV (ex(ΔNef-HIV)) or uninfected (ex(HIV-)) (15 μg/ml of exosomal protein except when 4 μg/ml is indicated, 48h) on the abundance and localization of lipid rafts (red) and TLR4 (green) in RAW264.7 macrophages. Scale bars 10 μm; **G**–Quantitation of the effect of exosomes (as in B)) on the total abundance of TLR4 in cells. Mean ± SEM are shown; *p<0.05, **p<0.01 *versus* exGFP and ex(HIV-). **H**–Quantitation of the effect of exosomes (as in B)) on the abundance of TLR4 in lipid raft regions. Mean ± SEM are shown; **p<0.01 *versus* exGFP and ex(HIV-/-); **J**–The effect of exNef, exGFP and exosomes isolated from plasma of subjects infected with HIV (ex(WT-HIV)) or infected with Nef-deficient strain of HIV (ex(ΔNef-HIV)) or uninfected (ex(HIV-)) (15 μg/ml of exosomal protein except when 4 μg/ml is indicated, 48h) and stimulation with 100 ng/ml LPS on secretion of TNFα by RAW264.7 macrophages over 1 h; Mean ± SEM are shown; **p<0.01, ***p<0.001; **I**–The effect of exNef, exGFP and exosomes isolated from plasma of subjects infected with HIV (ex(WT-HIV)) or infected with Nef-deficient strain of HIV (ex(ΔNef-HIV)) or uninfected (ex(HIV-)) (15 μg/ml of exosomal protein except when 4 μg/ml is indicated, 48h) and stimulation with 100 ng/ml LPS on secretion of IL-6 by RAW264.7 macrophages over 1 h; Mean ± SEM are shown; **p<0.01, ***p<0.001; **K**—Western blot for the indicated amounts of rNef and ex(WT-HIV) (μg of exosomal protein).

To further elucidate effects of Nef in the context of HIV infection, we isolated exosomes produced by human MDM either uninfected (Mock) or infected with WT HIV (exHIV) or Nef-deficient HIV (exΔNefHIV). MDM (from 6 different donors) were incubated with isolated exosomes equalized by RT activity in the presence of fusion inhibitor T-20 (to prevent infection by the virus present in exosome preparation) for 48 h; exosomes from uninfected cells were added at the exosome protein concentration equal to exHIV. The abundance of lipid rafts on cells (Fig 8A) as well as the amounts of TNFα and IL-6 secreted into the medium after stimulation with 1 ng/ml of LPS (Fig 8B and 8C) were significantly elevated in cells treated with exHIV as compared to cells treated with exosomes from uninfected cells or exΔNefHIV.

Next, we took advantage of availability of plasma from patients infected with Nef-deficient strain of HIV (ΔNef-HIV) [22] and compared the effects of exosomes isolated from plasma of these patients and patients infected with WT HIV or uninfected subjects. Plasma samples from subjects from each of the three groups (6 subjects in ΔNef-HIV infected group and 4 subjects in each of the two other groups) were pooled, exosomes isolated and tested in the experiments with RAW 264.7 murine macrophages. Murine macrophages could not be infected with HIV that might be present in the exosome preparations, so no fusion inhibitor was necessary. Abundance of rafts (Fig 8D and 8E), the abundance of TLR4 in the cells (Fig 8F and 8G) or in the lipid rafts (Fig 8F and 8H), and secretion of TNFα (Fig 8J) and IL-6 (Fig 8I) were similarly elevated in cells incubated for 48 h with exosomes produced by Nef-expressing HEK293 cells (exNef) or isolated from plasma of WT HIV infected subjects (ex(WT-HIV)) when compared to cells treated with the same concentration of exosomes produced by GFP-expressing HEK293 cells (exGFP) or isolated from plasma of ΔNef-HIV infected (ex(ΔNef-HIV)) or uninfected (ex(HIV-)) subjects. Concentration of Nef in ex(WT-HIV) was estimated as 0.1 ng Nef per 1 μg of exosomal protein (Fig 8K). These findings demonstrate that Nef-containing extracellular vesicles produced by HIV-infected cells modify lipid rafts and induce pro-inflammatory response.

## Discussion

In this study, we investigated how exosomes containing the HIV protein Nef alter cellular functions and cause inflammation associated with HIV infection. The main findings of this study can be summarized as follows: (*i*) Nef-containing exosomes (exNef) rapidly and effectively deliver Nef to macrophages; (*ii*) exNef affect cellular cholesterol metabolism (inhibit the abundance of ABCA1 and reduce cholesterol efflux) similar to the effect of HIV infection or recombinant Nef, but the effect of exNef was several orders of magnitude more potent than of recombinant Nef; (*iii*) exNef increase the abundance of lipid rafts through impairment of activation of Cdc42 followed by re-organization of actin cytoskeleton; (*iv*) exNef potentiate inflammatory responses *in vitro* and *in vivo*, likely through the effects on lipid rafts followed by activation of ERK1/2, activation of NLRP3 inflammasome and secretion of pro-inflammatory cytokines; (*v*) injection of exNef *in vivo* causes monocytosis, increases the abundance of rafts in monocytes, reduces ABCA1 abundance in several tissues, increases inflammatory cytokine production and causes hyperalphalipoproteinemia; (*vi*) *ex vivo*, exosomes isolated from HIV-infected cells or from plasma of HIV-infected subjects, but not from ΔNef-HIV infected cells or subjects, increase lipid rafts, TLR4 recruitment to rafts and inflammatory response in macrophages. The fact that Nef-containing exosomes produced by Nef-transfected HEK293 cells, HIV-infected MDMs and isolated from plasma of HIV-infected donors had similar effects on lipid rafts independently of the source, and exosomes not containing Nef did not show these effects, is a strong indication that the cause of these effects is in fact Nef, as opposed to other exosome constituents.

**Fig 9:**
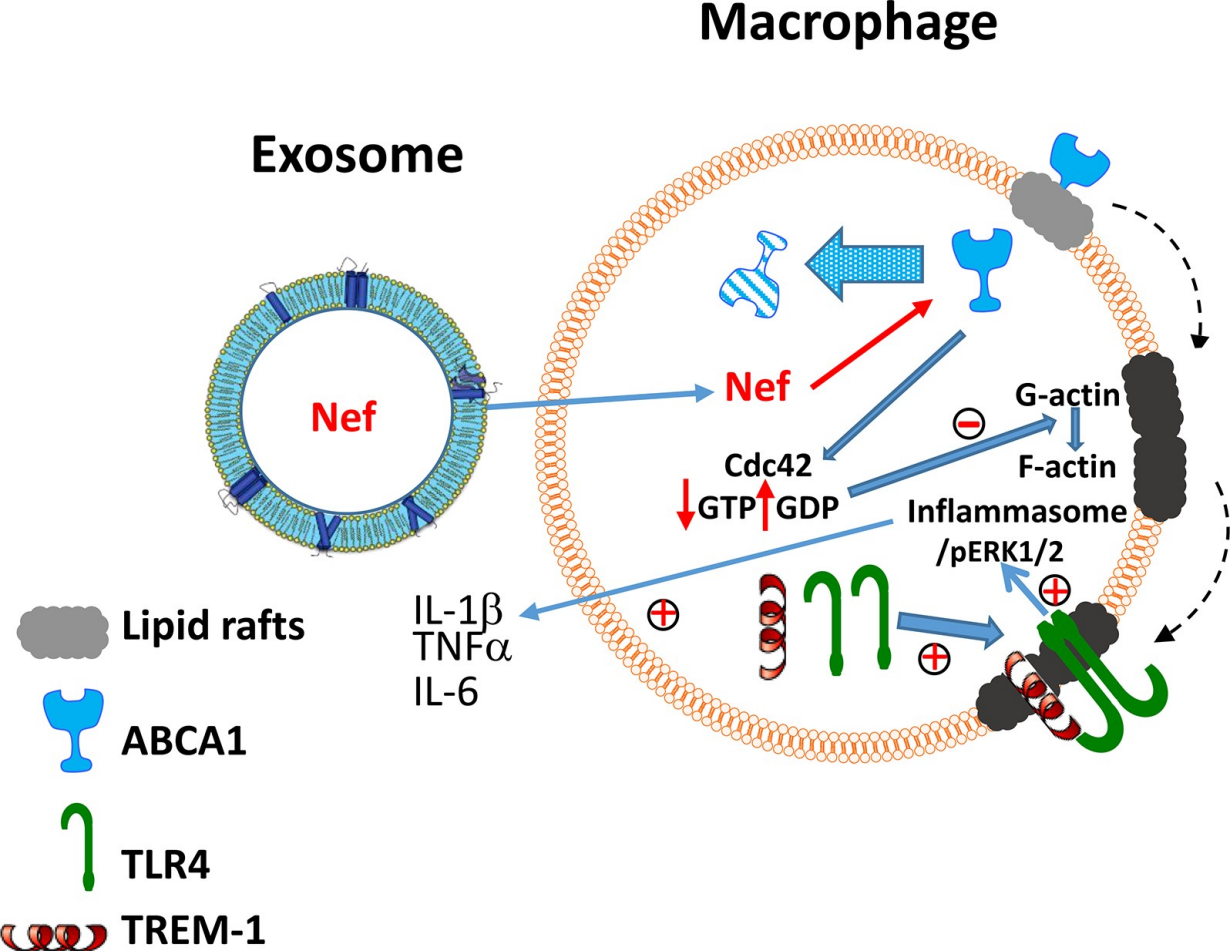
The proposed mechanism of the effect of exNef on inflammation. Nef released in exosomes from HIV–infected cells is taken up by bystander cells where it reduces the amount of ABCA1 by previously described mechanisms: displacement of ABCA1 from rafts with subsequent degradation of ABCA1 and preventing interaction of newly synthesized ABCA1 with calnexin, also followed by its degradation. Reduction of ABCA1 inhibits activation of Cdc42, which in turn decreases formation of filamentous actin enhancing formation of lipid rafts. Increase in lipid raft abundance leads to recruitment into rafts of TREM-1 and TLR4, leading to the activation of TLR4, phosphorylation of ERK1/2, activation of inflammasomes and stimulation of secretion of pro-inflammatory cytokines.

We propose the following mechanism of the effect of Nef on inflammation (Fig 9). 1. Nef released in exosomes from HIV–infected cells is taken up by bystander cells where it reduces the amount of ABCA1 by previously described mechanisms: displacing ABCA1 from rafts with subsequent degradation of ABCA1 [12] and preventing interaction of newly synthesized ABCA1 with calnexin, also followed by its degradation [36]. 2. Reduction of ABCA1 inhibits activation of Cdc42, which in turn decreases formation of filamentous actin enhancing formation of lipid rafts [34]. Several studies reported activation of Cdc42 by Nef [37, 38]. However, these studies investigated the effects of intracellularly produced Nef in T-lymphocytes, cells that express very little ABCA1 [12], and this activity of Nef may be independent to that in our study. 3. Increase in lipid raft abundance leads to recruitment into rafts of TREM-1 and TLR4, leading to the enhanced activation of TLR4, phosphorylation of ERK1/2, activation of NLRP3 inflammasome and augmented secretion of pro-inflammatory cytokines.

These findings are consistent with a suggestion that Nef-containing exosomes constitute an important factor in pathogenesis of several metabolic co-morbidities of HIV disease. Nef is dispensable for HIV replication *in vitro* [39], but patients infected with Nef-deficient HIV strain develop very mild, if any, HIV disease [40], implying that the main pathogenic function of Nef is systemic rather than intracellular [16]. Recombinant Nef injected into *apoe*^*-/-*^ mice fed a high-fat diet enhanced atherosclerosis and caused hypoalphalipoproteinemia and hypertriglyceridemia characteristic to HIV infection [41]. We propose that Nef creates a metabolic milieu that is favourable for HIV replication at the same time causes metabolic complications in the host. Cholesterol and lipid rafts play important role in HIV biology (for review see [10]), impairment of cholesterol metabolism and overabundance of rafts would benefit the virus, but at the same time may exacerbate co-morbidities that have impairment of cholesterol metabolism and inflammation as elements of their pathogenesis. These considerations firstly apply to atherosclerosis, a pathology responsible for a range of cardiovascular co-morbidities of HIV disease. Reduction in ABCA1 and consequently of cholesterol efflux, as well as increased abundance of TREM-1 in rafts, are recognized factors promoting accumulation of cholesterol in macrophages and inflammation [29, 42], two key elements in pathogenesis of atherosclerosis. Given that Nef-containing exosomes can be released from infected cells and have a considerable potency to affect cholesterol metabolism in uninfected cells, they can potentiate cholesterol accumulation and inflammatory response not only in HIV-infected, but in all macrophages, contributing significantly to the risk of atherosclerosis. Moreover, the effects of Nef on cholesterol metabolism in other cells involved in pathogenesis of atherosclerosis that cannot be infected with HIV, such as endothelial and smooth muscle cells, may also contribute to this co-morbidity.

Another co-morbidity likely related to the effects of Nef on cholesterol metabolism is HIV-associated neurocognitive disorder (HAND). HIV infects macrophages, glial cells and astrocytes, but does not infect neurons, which play a key role in neurodegenerative disorders, nor endothelial cells, responsible for the integrity of blood-brain barrier [43]. Recently, Raymond and Khan [9, 17] in a series of elegant studies demonstrated the role of Nef-containing exosomes in pathogenesis of HAND, however, direct toxicity of Nef was suggested as a putative mechanism. The role of cholesterol metabolism and of lipid rafts in pathogenesis of neurodegenerative diseases is firmly established [25, 44], so the effects of Nef, through regulating lipid rafts directly and/or through potentiating inflammation, may contribute, if not drive, neurodegeneration caused by HIV infection.

Pathogenesis of several other co-morbidities of HIV infection may also involve impairment of cholesterol metabolism by Nef. Haematological co-morbidities of HIV infection were, on the one hand, attributed to the action of Nef [45], and, on the other hand, have cholesterol metabolism in the centre of their pathogenesis [46]. The role of ABCA1 and cholesterol metabolism in pathogenesis of diabetes, a frequent co-morbidity of HIV infection [47], is also well established [48]. The effects of Nef on these and other HIV co-morbidities may be direct, targeting cholesterol metabolism-related elements of their pathogenesis, but also indirect, through potentiating inflammation, which in turn has impairment of cholesterol metabolism and enhanced formation of lipid rafts among the key elements of pathogenesis [26].

Nef-containing exosomes were found in plasma of patients receiving antiretroviral treatment and having no detectable viral load [9, 17, 49]. Most likely, the origin of these exosomes is a small number of HIV infected cells residing in HIV reservoirs [5, 50]. Secretion of exNef from infected cells can occur despite effective HIV suppression: current antiretroviral drugs affect every step of the HIV life cycle except transcription and translation, thereby allowing production and release of viral components such as Nef [7, 8]. Interestingly, Nef itself is a potent activator of microvesicle exocytosis, and mechanism of release of Nef-containing exosomes from infected cells has been recently described [9, 51]. Delivery by exosomes, however, is not the only mechanism behind the bystander effects of Nef: transfer of Nef to uninfected cells by trogocytosis has been recently demonstrated [51].

It must, however, be recognized that the broad clinical relevance of our findings remains unclear. While the results of the experiments on the effects of exosomes from pooled plasma samples of few HIV-infected subjects are consistent with the proposed mechanisms, they provide only limited evidence for the importance of these mechanisms in clinical setting, especially in the context of ART treatment. A clinical study relating clinical endpoints to biological activity of exosomes isolated from plasma of HIV-infected subjects treated with different regimens of ART and with different levels of exNef in their blood is required to convincingly address this question.

In conclusion, Nef released from HIV-infected cells in exosomes may be responsible for systemic impairment of cholesterol metabolism in cells not infected by HIV, causing inflammation and contributing to multiple co-morbidities of HIV disease.

## Methods

### Cells

Mouse macrophage cells RAW264.7 (ATCC, Manassas, VA) were grown in RPMI-1640 medium supplemented with 10% foetal bovine serum (FBS) (Gibco).

Human monocyte cells THP-1 (ATCC, Manassas, VA) were grown in RPMI-1640 medium supplemented with 10% FBS. To differentiate cells into macrophages Phorbol Myristate Acetate (PMA) was added to the media at concentration 100 ng/ml for 72 hours.

Bone marrow derived macrophages (BMDM) were isolated from tibia and femur bones of 6–8 week-old C57Bl/6 male mice as described by Shrestha et al [52]. In brief, bone marrow was flushed out from bones with IMDM containing 10% FBS. Cells were spun, pellet washed and incubated for 10 min in red blood cell lysis buffer (155 mM NH_4_Cl, 10 mM KHCO_3_, 0.1 mM EDTA, pH 7.3). Cells were centrifuged and pellet was resuspended in IMDM containing 10% FBS and 15% of L929 cell conditioned media. Cells were plated in Petri dishes (BD) and cultured for 5 days.

Human monocyte-derived macrophages were prepared from normolipidemic donor blood (obtained from New York Blood Bank) by plastic adherence and differentiated in the presence of 50 ng/ml M-CSF for 7 days.

Human embryonic kidney cells 293T (HEK293T, ATCC, Manassas, VA) were grown in DMEM supplemented with 10% exosome-depleted FBS.

SVEC4/VCAM-1 cells (a kind gift or Dr Alan Remaley, NHLBI) were grown in RPMI-1640 medium supplemented with 10% FBS.

### Antibodies

Primary antibodies used for the Western blotting analysis were:

1. Monoclonal mouse anti-Abca1 antibody (Abcam, #ab18180). This antibody was characterized in our previous studies [30, 53].
2. Monoclonal mouse anti-α1 sodium potassium ATPase antibody (Abcam, #ab7671). This antibody was characterized in our previous studies [53].
3. Monoclonal mouse anti-Cdc42 antibody (BD Transduction Laboratories, #610929). This antibody was characterized in our previous studies [53].
4. Monoclonal mouse anti-dynamin-2 antibody (BD Transduction Laboratories, #610248). This antibody was characterized in our previous studies [53].
5. Monoclonal mouse anti-Alix antibody (Novus biologicals, NB100-65678).
6. Monoclonal mouse anti-Nef antibody was obtained through the NIH AIDS Reagent Program, Division of AIDS, NIAID, NIH: Anti-HIV-1 SF2 Nef Monoclonal (EH1, #3689).
7. Monoclonal mouse anti ERK1/2 antibodies (Merck Millipore, #05–1152)
8. Monoclonal rabbit anti Phospho-p44/42 MAPK (Erk1/2), (Cell Signalling Technology, #4370)
9. Anti-pro Caspase-1 + p10 + p12 antibody, (Abcam, #ab179515)

Secondary antibodies used for the Western blotting analysis were:

1. Horseradish peroxidase-conjugated anti-mouse IgG antibody, (Sigma-Aldrich, #A9044).
2. Biotin conjugated anti-mouse IgG antibody (Sigma-Aldrich, #B8520).
3. Horseradish peroxidise-conjugated anti-rabbit IgG antibody (Cell Signalling Technology, #7074S).
4. VeriBlot for IP Detection Reagent (HRP) (Abcam, #ab131366)

Primary antibodies used for confocal microscopy were:

1. Polyclonal anti- ABCA1 antibody (Novus Biologicals, #NB400-105). This antibody was characterized in our previous studies [12, 53].
2. Monoclonal anti-ABCA1 antibody (Abcam, #ab18180). This antibody was used in co-localisation images of ABCA1 and rafts.
3. Monoclonal rat anti-TREM1 antibody (R&D Systems, #MAB1187)
4. Monoclonal mouse anti-TLR4 antibody (Abcam, #22048)

Secondary antibodies used for confocal microscopy were:

1. AlexaFluor 633-labelled anti-rabbit IgG antibody (Invitrogen, #A-21070).
2. AlexaFluor 546-labeled anti-mouse IgG antibody (Invitrogen, A111030)
3. AlexaFluor 546-labeled anti-rat IgG antibody (Invitrogen, A11081)

#### Transfections

RAW264.7 macrophages were transfected with constitutively active Cdc42 plasmid (pcDNA3-EGFP-Cdc42(Q61L), Addgene plasmid # 12600) [35]. Briefly, cells were seeded in antibiotic-free RPMI-1640 media supplemented with 10% FBS. Transfections were performed 18 h later when cells were 70–80% confluent. Transfections were done using Lipofectamine LTX with Plus Reagent (Invitrogen) according to manufacturer’s protocol.

HEK293 cells were transfected either with HIV-1 Nef_SF2_WT (kind gift of Dr Matija Peterlin) or control pEGFP- C1 plasmid (Clontech) using Lipofectamine LTX with Plus Reagent (Invitrogen) according to manufacturer’s protocol. Average transfection efficiency was 80%.

### Exosome isolation and purification

Transfected HEK293 cells were grown in DMEM supplemented with 10% heat-inactivated exosome-depleted FBS. Exosome-depleted FBS was prepared by ultracentrifugation for 2 h at 4°C, 100,000 x g using Beckman Coulter Optima MAX-TL centrifuge. Forty eight hours post transfection medium from cell culture was collected and exosomes were isolated as described by Rider et al [54]. Briefly, exosome-containing medium was pre-cleared from cellular debris by low speed centrifugation (2000 x g) for 30 min, mixed in equal volume of 16% Polyethylene glycol/1M NaCl (Sigma Aldrich), incubated at 4°C for 18 h on a rotating platform and centrifuged at 4°C, 4000 x g for 60 min. Pellet was collected, resuspended in PBS and spun again for 2 h at 4°C, 100,000 x g. Final pellet was resuspended in PBS, aliquoted and frozen at -80°C. Total protein content in exosome samples was estimated by Bradford assay after dilution in RIPA buffer and boiling for 3 minutes.

In some experiments exosomes were labelled with PKH67 Green Fluorescent Dye. PKH67 Green Fluorescent Cell Linker Kit for General Cell Membrane Labelling (Sigma-Aldrich) was used according to the manufacturer's protocol, except that washing procedure was modified according to Lässer et al [55]. After incubation with stain, exosomes were transferred to 300 kDa Vivaspin filters (Sartorius Stedim Biotech GmbH, Gottingen, Germany) and centrifuged at 4,000 × g. Samples were washed at least 3 times with 5 ml of PBS until there was no fluorescence in the wash-through before being transferred to new 300 kDa Vivaspin filters and washed twice with serum free RPMI.

### Electron microscopy and assessment of exosome size

Isolated exosomes were resuspended in 0.1 M sodium cacodylate buffer pH 7.2 to a required concentration, and 5μl was placed onto formvar carbon coated 200 mesh copper grids (Electron Microscopy Sciences, Hatfield, PA) for 1 min and removed. The grids were then fixed with 4% glutaraldehyde in sodium cacodylate buffer for 5 min and washed. They were stained with 2% aqueous uranyl acetate for 1 min, and imaged with a FEI Talos F200X Transmission Electron Microscope (Brno, Czech Republic) at 80 KV. The size of exosomes was ascertained from electron microscopy images using Image J software and calibrated magnification bars on the images.

### Experiments with exosomes from HIV-infected cells

To generate exosomes from HIV-infected cells, monocyte-derived macrophages were infected with Nef-positive and Nef-negative CCR5-tropic viruses (2x10^6^ cpm of RT activity per 10^6^ cells) pBRNL4.3_92BR020.4(R5)nef+_IRES_GFP and pBRNL4.3_92BR020.4(R5)nef-_IRES_GFP [56], respectively. Virus replication was followed by assaying reverse transcriptase activity in culture supernatants, and when it reached 1,200 cpm/μl (12 days post infection in culture infected with Nef-positive virus and 15 days post infection in culture infected with Nef-negative virus) the culture supernatants were collected and extracellular vesicles were isolated as described above.

Preparations of extracellular vesicles equalized by RT activity were added for 48 h to MDM from 6 different donors in the presence of fusion inhibitor T20 (1 μg/ml). At the end of incubation, cells were separated into two aliquots: one was analyzed for lipid rafts by staining with fluorescently-tagged CTB and flow cytometry, another was stimulated with 1 ng/ml of LPS and after 24 h was analyzed for production of IL-6 and TNFα as described below.

### Cell surface protein biotinylation and Western blots

Cells were lysed with RIPA buffer, protein concentration in lysates estimated by Bradford assay followed by SDS-PAGE and transfer of proteins to PVDF membrane. To assess abundance of the cell-surface ABCA1, cells were washed three times with ice cold PBS and biotinylated with Sulfo-NHS-SS-Biotin as described previously [12, 53]. Biotinylation reaction was quenched by washing cells twice with 50 mM Tris, 150 mM NaCl, 0.1 mM EDTA. Cells were lysed with RIPA buffer and biotinylated proteins were purified by incubating for 2 h at 4°C with High Performance Streptavidin Sepharose (Thermo Fisher Scientific). Bound biotinylated proteins were removed from resin by 30 min incubation in loading buffer supplemented with 50 mM DTT at 50°C and centrifugation. Supernatants were subjected to SDS-PAGE, proteins were transferred to PVDF membrane and probed.

### Cholesterol efflux

Cholesterol efflux was measured as described previously [53, 57]. In brief, cells were labelled by incubation in serum-containing medium supplemented with [^3^H]cholesterol (75 kBq/ml, American Radiolabelled Chemicals for 48 h. Cells were washed and incubated for 18 h in serum-free medium in the presence of LXR agonist TO-901317 (final concentration, 4 μM). Human apoA-I (kind gift from CSL Behring) was added to the final concentration of 30 μg/ml and cells were incubated for 2 h at 37°C. The efflux was calculated as a proportion of radioactivity moved from cells to medium; non-specific efflux (i.e. the efflux to the medium without acceptor) was subtracted.

### Confocal microscopy

For confocal microscopy studies cells were seeded in μ-slide 8-well chambers (Ibidi, USA), grown overnight and used for further treatments. Cells were then washed with ice-cold PBS and fixed with 4% paraformaldehyde for 15 min at room temperature. Cells were permeabilised using treatment with 0.1% triton X-100 for 10 min at room temperature, washed and blocked with 10% goat serum for 30 min. Cells were then stained with a corresponding antibody. Images were collected using a Nikon A1r+ confocal microscope equipped with 60x Water Immersion objective (Nikon 60x or 40x Plan Apo VC, WI NA 1.2). Images were collected with the 405, 488 and 568 lasers sequentially to minimise bleed through. Co-localisation was quantitated using Coloc2 feature in Fiji software on at least 50 to 100 cells. The abundance of lipid rafts was assessed using the Vybrant lipid raft labelling kit (Life Technologies) according to manufacturer’s instructions. Briefly, cells were incubated with Alexa Fluor 488-labeled CTB for 10 min on ice, washed, stabilized by incubation with anti-CTB antibody for 15 min at 4°C and fixed with 4% paraformaldehyde for 15 min at room temperature. F-actin was visualized with phalloidin staining according to manufacturer’s instructions (Abcam, ab176753). Briefly, cells were fixed with formaldehyde, washed with PBS, permeabilised with 0.1% Triton X-100 for 3–5 minutes and then washed again with PBS. Cells were incubated with phalloidin-conjugate working solution for 20–90 minutes and washed to remove excess stain.

### Inflammasome activation

Murine BMDM were seeded in 10% FBS IMDM supplemented with 10% L929 conditioned media. The next day exosomes were added and incubated 48 h. To prime inflammasomes, cells were incubated with LPS (100 ng/ml) for 4h. To stimulate release of Il-1β, cells were incubated with Nigericin (5 μM) for 3h. Cell culture supernatants were collected to measure concentration of Il-1β by ELISA and abundance of p10 fragment of caspase-1 by IP. Cell lysates were collected for estimation of procaspase-1 by Western blot.

### qRT-PCR

Cells were grown in a 12 well plate and treated with exosomes for indicated periods of time. Total RNA was extracted from these cells using TRIzol reagent from Ambion (Life Technologies, #15596018). Two μg of RNA was DNAse treated using a kit from Ambion, (#2222) from which cDNA was made using High capacity cDNA reverse transcription kit from Applied Biosystems (#4368814). Primers for mouse ABCA1 and Nef were from Taqman Gene Expression Assays (Applied Biosystems #448892). The assay was performed on a 7500 fast system from Applied Biosystems in triplicate and the relative amount of mRNA was calculated using the comparative Ct method with 18s rRNA as the housekeeping gene.

### RNA sequencing

For RNA isolation, exosomes were prepared using PEG precipitation, resuspended in cold PBS and centrifuged in Optima XPN-100 Ultracentrifuge (Beckman Coulter) at 100,000 x g, 4°C for 75 min. After ultracentrifugation, pellets were resuspended in 200 μl of ice cold Exosome Resuspension Buffer (Total Exosome RNA & Protein Isolation Kit, Invitrogen) and stored at -80°C. RNA was isolated using the manufacturer’s protocol. For preparation of a library of RNAs longer than 200 nucleotides, TruSeq Stranded mRNA kit (Illumina) was used. Library of miRNAs was prepared using the TruSeq small RNA kit (Illumina). Both libraries were sequenced on the Illumina NextSeq 500 machine. Single-end reads of 75 bp were sequenced for the long RNAs and 50 bp single-end reads—for the small RNAs.

### Cdc42 activity assay

Cdc42 activity assay was done using G-LISA Cdc42 Activation assay (Cytoskeleton, Inc). This assay detects the amount of GTP-bound (active) Cdc42. Briefly, exosomes treated RAW264.7 cells were washed with PBS and activated with bradykinin (Sigma-Aldrich) (100 ng/ml) in serum free RPMI for 4 minutes. Cells were washed with warm serum-free RPMI, lysed and snap frozen. Protein concentration was estimated and samples were used in G-LISA according to manufacturer’s protocol.

### ELISA for cytokines

TNFα and IL-6 in the supernatant of human monocyte derived macrophages treated with Nef or control exosomes were measured by commercial ELISA (R&D Systems) according to manufacturer’s protocol. Briefly, for cellular experiments MDM were stimulated for 24 h with 1 ng/ml LPS, supernatants were collected and used in ELISA at several dilutions. Cells were lysed and protein concentration measured by Bradford assay (Bio-Rad). Final cytokine concentration was normalized to cellular protein content.

TNFα and IL-6 in murine plasma were measured by ELISA assay from Invitrogen according to manufacturer’s instructions.

### Bio-assay for cytokines

SVEC4/VCAM-1 cells were used to assess the effect of secreted cytokines on VCAM-1 expression [58]. Briefly, RAW 264.7 cells were treated with exosomes for 48h, activated with LPS (0.1 μg/ml) for 12 h. Media was collected and spun at 100,000 x g for 70 minutes to remove exosomes. Conditioned medium was added to SVEC4/VCAM-1 cells and incubated for 4h. Luciferase activity was measured using Bright-Glo Luciferase Assay System (Promega).

### Apoptosis and necrosis assay

The viability of RAW264.7 cells treated with exosomes was tested using a Cell Death Detection ELISA kit (Roche). Briefly, cells were treated with exosomes for 48h, media was collected, spun and tested for necrosis. Cells were tested for apoptosis according to kit manufacturer’s instructions.

### Lipidomics

Lipidomic analysis was performed as described previously [30]. Lipid raft fractions were combined and sonicated on ice; lipids were extracted using a, single phase CHCl_3_:CH_4_OH method. The analysis was performed by liquid chromatography electrospray ionization-tandem mass spectrometry (LC ESI-MS/MS) using a Agilent 1200 liquid chromatography system (Agilent Technologies) and Applied Biosystems API 4000 Q/TRAP mass spectrometer with a turbo-ion spray source (350°C) and Analyst 1.5 and MultiQuant data systems (AB SCIEX). Lipid concentrations were calculated by relating the peak area of each species to the peak area of the corresponding internal standard (one per group).

### Animal studies

C57BL/6 mice were purchased from Jackson Laboratories and colonies were maintained at AMREP. Mice were housed in a normal light and dark cycle and had ad libitum access to food (normal chow) and water. C57BL/6 mice were administered either exNef (2μg, I.V.) or control (exGFP) exosomes (2μg, I.V.), 3 times a week, for a period of 2 weeks. At the end of the experiment mice were euthanized and blood was collected via cardiac puncture into EDTA tubes, which were immediately placed on ice. Liver and spleen were excised and peritoneal macrophages were collected after washing peritoneal cavity with PBS.

For flow cytometry analysis, red blood cells were lysed (BD pharm Lyse; BD Biosciences), and white blood cells were centrifuged, washed, and resuspended in HBSS (0.1% BSA w/v, 5 mM EDTA). Cells were stained with a cocktail of antibodies against CD45-PB, Ly6-C/G-PerCP-Cy5.5 (BD Biosciences), CD115-PE (eBioscience) and CTxB-FITC. Monocytes were identified as CD45^hi^CD115^hi^ and further subdivided into Ly6-C^hi^ and Ly6-C^lo^. CTB binding was measured as mean fluorescence intensity (MFI). Samples were run on the LSR Fortessa, and analysed using FlowJo. Plasma total cholesterol, HDL cholesterol and triglyceride content were measured by colorimetric assays from Wako (Japan) according to manufacturer’s instructions.

### Human studies

A cohort of subjects infected with Nef-deficient strain of HIV has been described in our previous publication [22]. Four subjects infected with WT HIV were selected from a current study. The study details will be described elsewhere, but briefly all subjects were normolipidemic adult males 41±8 yo, not receiving antiretroviral therapy, average CD4+ T cell count of 470±282 cells/μl, and viral load of 8±10 x 10^4^ copies/ml. Plasma from uninfected subjects was obtained from healthy volunteers. Plasma samples from each group were pooled before exosomes were isolated as described above with following modifications: final concentration of PEG was reduced to 5% and incubation time to 2h to avoid co-precipitation of plasma lipoproteins. with Isolated exosomes (final concentration 15 μg/ml of exosomal protein, unless stated otherwise) were incubated for 48 h with murine macrophages RAW 264.7 and rafts and TRL4 were visualized and quantitated using confocal microscopy as described above.

### Ethics Statement

All participants provided written informed consent and the study was approved by the Alfred Hospital Human Research and Ethics Committee (#377/10). Plasma samples of subjects infected with Nef-deficient HIV used in this study were from the previous studies [22, 59]; original human ethics approvals permitted for the extension of analysis of the collected samples. All participants were adults.

All animal experiments were approved by the Alfred Medical Research Education Precinct (AMREP) animal ethics committee (#6553) and conducted in accordance with the Australian code of practice for the care and use of animals for scientific purposes as stipulated by the National Health and Medical Research Council of Australia. All mice were housed in a normal light and dark cycle and had *ad libitum* access to food and water. Mice were randomly assigned to treatment and end-point analysis was blinded.

### Statistics

All data is shown as mean ± standard error of means (SEM) unless stated otherwise. In box plots the horizontal line in each box indicates the median value with its height representing the 25–75th percentiles; box plot whiskers represent the extreme maximum or minimum data points. Statistical significance of the differences was assessed in SigmaStat or GraphPad Prism software packages by unpaired Student’s *t*-test or one-way ANOVA when data followed normal distribution or Mann-Whitney U test on ranks. When comparing the effects of exosomes on MDM from different donors, Group analysis and two-way ANOVA were used. The experiments were conducted in quadruplicates and repeated 2–5 times. When normalization of data was difficult, and the experiments were performed in multiplicates, representative experiments out of 2–3 similar experiments are shown.

## Supporting information

S1 Dataset MiRNA abundance in exosomes produced by Nef expressing *versus* mock-transfected cells. (XLSX)

S2 Dataset Results of the lipidomics analysis of rafts from cells treated with exNef *versus* exGFP. (XLSX)

S3 Dataset Results of the lipidomics analysis of plasma of mice injected with exNef *versus* exGFP. (XLSX)
